# The Role of Galectin-3 in Heart Failure—The Diagnostic, Prognostic and Therapeutic Potential—Where Do We Stand?

**DOI:** 10.3390/ijms241713111

**Published:** 2023-08-23

**Authors:** Beata Zaborska, Małgorzata Sikora-Frąc, Krzysztof Smarż, Ewa Pilichowska-Paszkiet, Andrzej Budaj, Dariusz Sitkiewicz, Grażyna Sygitowicz

**Affiliations:** 1 Department of Cardiology, Centre of Postgraduate Medical Education, Grochowski Hospital, 04-073 Warsaw, Poland; bzaborska@cmkp.edu.pl (B.Z.); msikora@cmkp.edu.pl (M.S.-F.); epilichowska@cmkp.edu.pl (E.P.-P.); abudaj@cmkp.edu.pl (A.B.); 2 Department of Laboratory Medicine, Medical University of Warsaw, 02-091 Warsaw, Poland; dariusz.sitkiewicz@gmail.com (D.S.); gsygitowicz@poczta.onet.pl (G.S.)

**Keywords:** galectin-3, heart failure, atrial fibrillation, fibrosis

## Abstract

Heart failure (HF) is a clinical syndrome with high morbidity and mortality, and its prevalence is rapidly increasing. Galectin-3 (Gal-3) is an important factor in the pathophysiology of HF, mainly due to its role in cardiac fibrosis, inflammation, and ventricular remodeling. Fibrosis is a hallmark of cardiac remodeling, HF, and atrial fibrillation development. This review aims to explore the involvement of Gal-3 in HF and its role in the pathogenesis and clinical diagnostic and prognostic significance. We report data on Gal-3 structure and molecular mechanisms of biological function crucial for HF development. Over the last decade, numerous studies have shown an association between echocardiographic and CMR biomarkers in HF and Gal-3 serum concentration. We discuss facts and concerns about Gal-3’s utility in acute and chronic HF with preserved and reduced ejection fraction for diagnosis, prognosis, and risk stratification. Finally, we present attempts to use Gal-3 as a therapeutic target in HF.

## 1. Introduction

**Figure 1:**
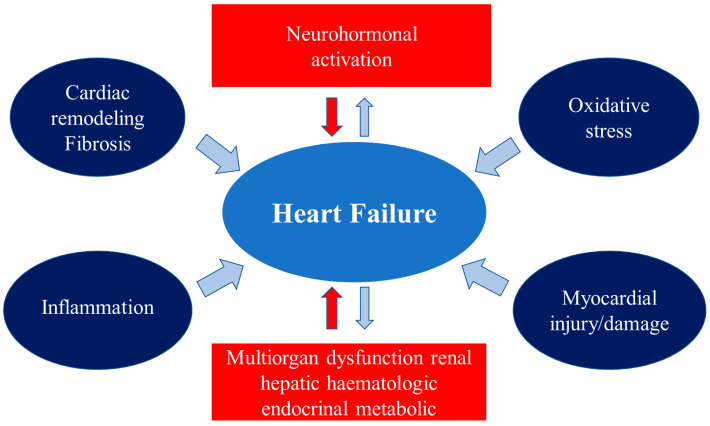
Major pathophysiological mechanisms leading to the onset and progression of HF.

Heart failure (HF) remains one of the most challenging medical conditions associated with high morbidity and mortality, despite the diagnostic and therapeutic efforts. HF is a clinical syndrome, not a single pathological diagnosis, with various stages and presentations, acute (AHF) and chronic. The main heart pathologies observed in HF include myocardial dysfunction (systolic, diastolic, or both), valve diseases and pericardium abnormalities, and heart rhythm and conduction disturbances, which lead to elevated cardiac pressures and/or inadequate cardiac output at rest and /or during exercise [1]. HF affects more than 64 million patients worldwide and its overall prevalence is increasing, reaching 1–3% of the general adult population in industrialized countries [2]. Risk stratification remains difficult, even though numerous prognostic markers of death have been identified [1,3]. Therefore, there is a growing interest in biomarkers, either biochemical or imaging-related. The fields of investigation are wide because the main pathophysiological pathways involved in HF are complex and multiorgan-associated (Figure 1) [4].

Galectin-3 (Gal-3) is the only chimera-type member of the lectin family, widely expressed in human tissues, acting as a galactoside-binding protein involved in many biological processes, such as controlling cell–cell and cell–matrix interactions, adhesions, proliferation, apoptosis, immunity, and inflammation [5]. In the pathology of HF, Gal-3 plays a biological role mainly through fibrosis and inflammation [6]. Gal-3 stimulates pathological remodeling and development of fibrosis, mainly by inducing fibroblast proliferation and collagen deposition, and, therefore, is called a “culprit” biomarker in HF in contrast to “bystanders” biomarkers, such as N-terminal pro-B-type natriuretic peptide (NT-pro BNP) or C reactive protein [7].

Higher concentrations of Gal-3 have been related to higher mortality in the general population and HF patients [4,8]. Since 2014, Gal-3 has been approved by the US Food and Drug Administration as a new biomarker for additive risk stratification in HF [9]. Moreover, Gal-3 measurements are recommended by the 2017 guidelines of the American Heart Association for risk stratification and prognosis assessment in HF patients [10]. However, in the guidelines recently released by the European Society of Cardiology and by AHA/ACC/HFSA, the recommendation for Gal-3 is not present [1,3]. Despite a large pool of interesting biomarker candidates, including Gal-3 besides natriuretic peptides, virtually none have succeeded in being applied in the clinical setting [11].

Nevertheless, the biological role and molecular mechanism of Gal-3 in HF are vigorously investigated and reported in the literature. This review is focused on recently published papers on the involvement of Gal-3 in HF, its role in pathogenesis, and clinical diagnostic and prognostic significance. We discuss the potential implication of Gal-3 as a molecular target for new promising therapies for the treatment of HF.

## 2. Galectins

### 2.1. Galectin Family

**Figure 2:**
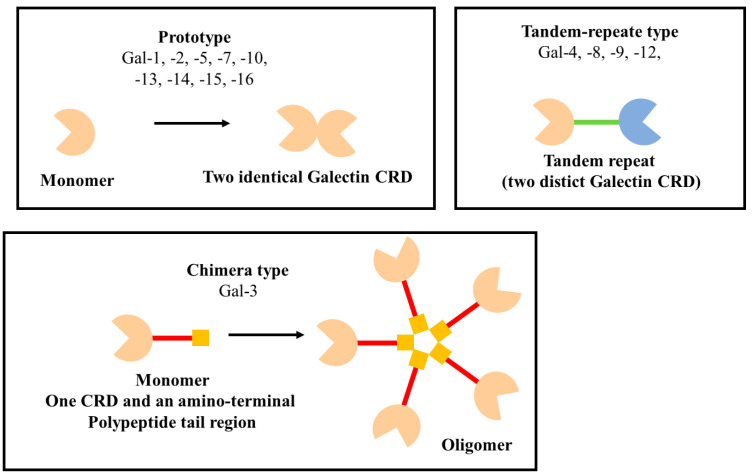
Structure of the galectin family members.

Galectins constitute a very important family of β-galactoside-binding lectins, playing a significant role in modulating “cell–cell” and “cell–matrix” interactions [12,13,14]. A common molecular structure of all galectins is the C-terminal recognition/binding domain (CRD), owing to which galectins bind to and regulate the activity of glycoproteins [15,16]. Highly conservative carbohydrate recognition domains occur as singles or doubles in mammalian galectins. They form complexes, which cross-link the glycosylated ligands [6,17,18,19]. Galectins have been divided into three subgroups, according to their CRD number and function (Figure 2): prototype galectins (galectin-1, -2, -5, -7, -10, -11, -13, -14, -15, and -16) contain a single CRD domain, which forms noncovalent homodimers, tandem-repeat galectins (galectin -4, -6, -8, -9 and -12) containing two CRD domains connected by a peptide link, and also galectin of chimera type (galectin-3), which is characterized by a single CRD and amino-terminal polypeptide tail region [13,17,18,20].

In the nomenclature of galectins, an important regularity has been maintained, concerning galectin numbering according to the order of their discovery. Galectins are ubiquitous in vertebrates, invertebrates, and protists [12]. Many galectin family members are detected mainly intracellularly, although some members can be found both inside and outside cells. Specific functions for particular galectins, agreeing at the same time with their intracellular location, have been ascribed to some types of galectins. Galectin-1 and -3 have been identified as redundant pre-mRNA splicing factors. It has been shown that Gal-3, -7, and -12 regulate cell growth and, also, apoptosis, being either anti-apoptotic or pro-apoptotic factors. At the same time, it has been demonstrated that Gal-3 and -12 regulate the cell cycle. Furthermore, the cellular functions on the cell surface can be also regulated by galectin signaling. Outside and on the surface of the cell, galectins interact with glycoconjugates, modulating cell–ECM and cell–cell interactions. In addition, galectins can participate in interactions between molecules in the cell membrane. In the cytosol and nucleus, galectins are mainly involved in glycan-independent protein/protein interactions as well as in signaling and splicing mRNA. Not all biological functions of galectins have yet been learned. Those that are known include, among other functions, an important role in the processes of development, tissue regeneration, or regulation of immune cell activity [12,13].

Galectins are important regulators of inflammatory and immune system responses. In fact, galectins are expressed in many inflammatory cells, e.g., macrophages [6,19]. Depending on the inflammatory environment, galectins promote either proinflammatory or anti-inflammatory reactions [14,17,18]. Specific molecular recognition of glycans on cell surfaces, mediated by galectins, revealed their immune activity against potential pathogens and parasites. Therefore, galectins, constituting a part of the inborn immune system of recognition of microorganisms, function as effectors binding to exogenous glycans, which are present on the surface of viruses, bacteria, fungi, and parasites [14].

### 2.2. Structue and Function of Galectin-3

Gal-3 with its unique structure, different than that of other galectin family members, is able to form pentamers. Gal-3 contains a proline/glycine-rich N-terminal domain through which it is able to form oligomers. It should be noted, however, that pentamer formation has been observed in DMSO, and so far, it is not certain that this process occurs in vivo. A high concentration of Gal-3 monomers contributes to the increase in the capacity and stability of ligand binding [15]. Galectin-3 is the only galectin of the chimera type with a molecular weight of 29–35 kDa and consists of 251 amino acid residues and 2 different domains. The N-terminal domain consisting of 120 amino acids contains a tandem repeatable short amino acid segment of proline, glycine, alanine, and tyrosine. Parts of the N-terminal domains are cross-linked with the C-terminal carbohydrate recognition domains. Although the C-terminal domain directly determines lectin activity, both the N-terminal and C-terminal domains are responsible for the full biological activity of galectin-3 [21]. Gal-3 is encoded by a single *LGALS3* gene, present in the human genome on chromosome 14, locus q21-22, and consisting of six exons and five introns of about 17 kilobases in length. The methylation status of the *LGALS3* promoter and elements such as CRE motifs, nuclear factor κB (NF-κB)-like loci, and GC boxes located on the Gal-3 promoter regulate the expression of Gal-3. Gal-3 also contains a special regulatory element called galig (galectin-3 internal gene), located in the second intron of the *LGALS3* gene [19]. Transcriptions of Gal-3 and internal galig promoter have common encoding sequences but utilize alternative reading frames. Galig is the cell death gene, encoding two proteins: cytoplasmic protein cytogaligin and mitochondrial protein mitogaligin. It is believed that mitogaligin interaction with cardiolipin causes a disturbance of the mitochondrial membrane, while cell death induced by galig can be prevented through overexpression of myeloid cell leukemia sequence protein 1 (MCL-1), which belongs to the anti-apoptotic Bcl-2 family [22]. Gal-3 transcription can be also suppressed by proteins such as Krüppel factor 3 (KLF-3), belonging to the family of zinc finger transcription factors [23], or it can be activated by other transcription factors, such as runt-related transcription factor 2 (RUNX2) [24].

**Figure 3:**
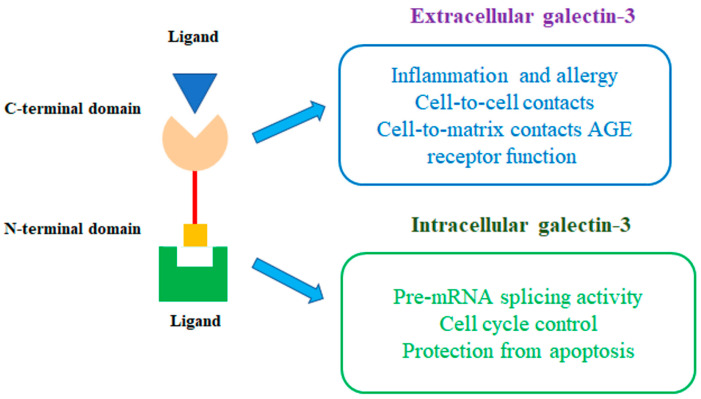
The intracellular and extracellular functions of Gal-3.

Gal-3 is expressed in the nucleus, cytoplasm, mitochondria, cell surface, and extracellular space. It is known that glycoproteins account for 50% to 70% of all proteins and are widely distributed in the nucleus, cytoplasm, cell membrane, and extracellular matrix. That allows Gal-3 to exert pleiotropic effects under pathological conditions. Owing to its expression, significantly and rapidly induced under pathological conditions, Gal-3 has gained a leading importance, compared with other galectins. Gal-3 plays an equal or even more important role than other galectins, in cell–cell interactions or cell–matrix interactions and participates in cell growth and differentiation, activation of macrophages, antimicrobial activity, angiogenesis, and apoptosis [19,25]. The extracellular and intracellular activity of Gal-3 is shown in Figure 3.

Extracellular Gal-3 mediates cell migration and adhesion, cell–cell interactions by binding to galactose-containing glycoproteins on the cell surface. Intracellular functions of Gal-3 have been extensively documented. In some cases, intracellular proteins with which the protein interacts and which possibly mediate these functions have been identified. Gal-3 can be phosphorylated at serines 6 and 12 and tyrosines 79, 107, and 118 by c-Abl [26,27].

Gal-3 has a common specificity for β-galactose residues, which are widely distributed on glycoproteins and glycosphingolipids. Gal-3 interacts with a plethora of proteins present in the cell membrane, in the extracellular matrix, in biological fluids, and intracellularly, with the possibility of the CRD-independent protein–protein interaction in the latter case [28] The Gal-3 CRD is physiologically suited to bind natural polysaccharide ligands. Natural ligands such as the disaccharides lactose and *N*-acetyllactosamine bind through an extended network of hydrogen bonds between their hydroxyl groups, the polar amino acids of Gal-3, and water molecules and relatively weak van der Waals interactions, hence providing low binding affinities [28].

Gal-3 also plays a vital role in the interaction between epithelial cells and the extracellular matrix. Cytoplasmic Gal-3 modulates cell survival due to its anti-apoptotic activity and, also, participates in the regulation of signal transduction pathways. Nuclear Gal-3 is associated with pre-mRNA splicing and gene expression and can therefore promote cell proliferation.

Similarly, as other galectins, Gal-3 has no secretory signal peptide for classic exocytosis using vesicles; therefore, it is located mainly in the cytoplasm and sometimes in the nucleus and mitochondria. After secretion into the extracellular space (through a nonclassic secretory pathway that circumvents the endoplasmic reticulum and Golgi complex) [29], Gal-3 can interact with cell surface receptors and glycoproteins to initiate transmembrane signaling pathways for various cell functions. Although it is known that many factors affect Gal-3 secretion, e.g., thermal shock, calcium ionophores, acylation, and phosphorylation [30], a detailed explanation of their mechanisms of action is still expected. Intracellular mechanisms, including phosphorylation reactions and importin-mediated mechanisms, seem to be engaged in nucleo-cytoplasmatic transportation of Gal-3 [31,32], while synexin-mediated mechanisms are suggested in Gal-3 translocation to the mitochondria [33].

It has been demonstrated that Gal-3 participates in many pathological processes, such as inflammation [19], fibrosis, cell–cell [34,35] or cell–matrix interactions [36], cell proliferation, and protection against apoptosis [19]. Moreover, many studies have shown that Gal-3 expression is detected in many pathological conditions, such as cardiac diseases [37,38,39,40,41,42,43], kidney diseases [44,45,46,47], diabetes [44,47,48], viral infections [49,50,51,52], autoimmune diseases [53,54,55,56], neurodegenerative disorders [57,58], and tumor development [32,33,35].

**Figure 4:**
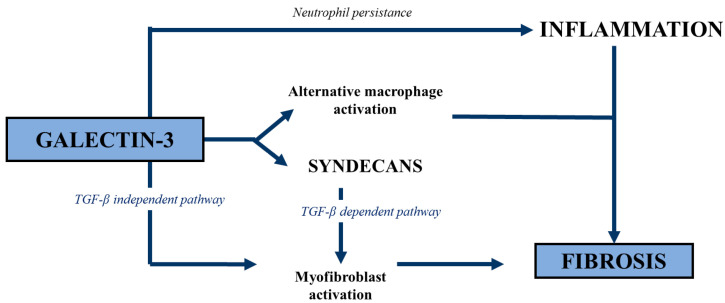
The role of galectin-3 in fibrosis.

Gal-3 is involved in the pathophysiology of heart failure [38], mainly due to its role in the remodeling of the heart ventricles [59,60]. The expression of Gal-3 in the heart is usually low, while in heart failure both its synthesis and secretion increase significantly [37,60,61]. Gal-3 initially plays a protective role in the heart through its apoptotic and anti-necrotic effects. However, a prolonged increased expression of Gal-3 leads to fibrosis and unfavorable remodeling of the damaged tissue [38]. The sites of Gal-3 binding are mainly located in the extracellular matrix, fibroblasts, and macrophages. At the site of injury, Gal-3 is secreted into the extracellular space and activates resting fibroblasts to matrix-producing fibroblasts. The role of Gal-3 in fibroblast activation includes increasing the expression of cytoskeleton proteins, synthesis of new matrix components, such as type I collagen, and inhibition of extracellular matrix component degradation through inhibition of expression of matrix metalloproteinases [38]. Moreover, it is suggested that Gal-3 may be engaged in both the development and regression of fibrosis [62]. The role of Gal-3 in fibrosis has been well established (Figure 4), and increased Gal-3 levels contribute to the activation of (myo)fibroblasts through a TGF-β-independent pathway and through a TGF-β-dependent pathway. Syndecans also play an important role, particularly through their effect on profibrotic signaling in cardiac fibroblasts and, possibly, through interactions with Gal-3. Moreover, Gal-3 can also affect the fibrotic pathway through the induction of an alternative activation (M2) in macrophages [63].

## 3. Acute Heart Failure

### 3.1. Prediction of Symptoms

As Gal-3 is secreted by activated macrophages and reflects myocardial fibrosis and remodeling, it could be used as a marker of clinical deterioration in high-risk patients. Previous studies suggested a potential role of Gal-3 in predicting symptoms and prognosis as a component of the multiparametric approach [64,65]. In animal models, early and late secretion of Gal-3 after myocardial infarction was observed. It has been proved in animal studies with the Gal-3 knockout mice model that Gal-3 is essential for wound healing after MI and that lack of Gal-3 leads to inappropriate scar formation and cardiac rupture [66]. Anti-apoptotic and anti-inflammatory activation by Gal-3 is responsible for this protective regulation in the early phase of ischemia [67,68]. In the later phase, Gal-3 leads to chronic inflammation, fibrosis, and adverse remodeling and increases the risk of HF development [69]. Clinical studies have also confirmed that elevated Gal-3 is related to LV remodeling [70], HF, and mortality after myocardial infarction [50,71,72].

Recent studies have shown Gal-3 as a predictor of HF development after acute ST-elevation myocardial infarction treated with primary percutaneous intervention [73].

### 3.2. Predictor of Prognosis

Gal-3 is an important prognostic marker in patients with acute HF. In a meta-analysis of 18 studies including 7057 AHF patients, higher serum Gal-3 was associated with higher risks of all-cause mortality (adjusted risk ratio [RR], 1.58; *p* < 0.001), mortality/HF rehospitalization (RR, 1.68; *p* < 0.001), and cardiovascular mortality (RR, 1.29; *p* = 0.04) but not HF rehospitalization (RR, 1.24; *p* = 0.25) [74]. In the randomized SHOCK-COOL trial, the prognostic role of Gal-3 was investigated in 40 patients with cardiogenic shock complicating acute myocardial infarction, who were randomly assigned to the mild therapeutic hypothermia 33 °C or control groups. Patients with lower serum Gal-3 concentration on the first day after admission demonstrated a higher risk of all-cause mortality at 30 days. Serum Gal-3 concentration on day 1 had a good predictive value for 30-day all-cause mortality with an area under the receiver operating characteristic curve of 0.696 (95% CI: 0.513−0.879), with an optimal cutoff point of less than 3.65 ng/mL [75].

A meta-analysis of 9217 patients conducted by Wu et al. revealed the prognostic significance of Gal-3 in chronic and acute HF patients. The diagnostic hazard ratios of Gal-3 in predicting mortality in chronic HF patients were 1.13 (95% CI: 1.07–1.21) and 2.17 (95% CI: 1.27–3.08) in AHF patients [76]. This predictive value was later confirmed by Yao et al. The authors in a study of patients with acute onset of HF, among other biomarkers, found Gal-3 to be a predictive marker of prognosis [77].

Gal-3 could be especially useful in some special populations with the highest risk of adverse events, such as patients with chronic renal dysfunction. However, these data have been ambiguous until now. Although Bansal et al. found no association between Gal-3 plasma concentration and incident HF or atrial fibrillation (AF) in an observational study of chronic kidney disease patients [78], further studies provided the opposite findings. Caravaca et al., in an observational, prospective, multicenter registry of 1201 patients hospitalized for acute HF, showed a negative correlation of Gal-3 with eGFR (rho = −0.51; *p* < 0.001). Higher Gal-3 serum concentrations were associated with higher mortality risk in the multivariate analysis after adjusting for eGFR and other prognostic variables [HR = 1.010 (95% CI: 1.001–1.018); *p* = 0.038]. However, the prognostic value of Gal-3 was restricted to patients with renal dysfunction [HR = 1.010 (95% CI: 1.001–1.019), *p* = 0.033] with an optimal cutoff point of 31.5 ng/mL, with no prognostic value in the group with preserved renal function [HR = 0.990 (95% CI: 0.964–1.017); *p* = 0.472] [79]. In another study of AHF patients, who were enrolled in the acute kidney injury neutrophil gelatinase-associated lipocalin evaluation of symptomatic EF study (AKINESIS) conducted by Horiuchi et al., the authors found more common acute kidney injuries and myocardial injuries in patients with Gal-3 serum concentration > 25.9 ng/mL. In multivariable analysis, higher Gal-3 concentrations were associated with 1-year mortality [80].

In another group of AHF patients from the AKINESIS study, who were categorized according to the body mass index (BMI), a positive correlation between Gal-3 serum concentration and BMI was found. However, Gal-3 was significantly associated with 30-day mortality or HF readmission only in patients with normal BMI (18.5–24.9 kg/m^2^) and showed no association with other BMI categories [81].

The serum concentration of Gal-3 in patients hospitalized with AHF depends not only on cardiac involvement but also on many other conditions, such as renal dysfunction, hepatic cirrhosis, pulmonary fibrosis, cancer disease, and obesity. The role of Gal-3 in the prognosis of patients with AHF needs to be estimated in further prospective studies.

## 4. Chronic Heart Failure

The use of Gal-3 as a biomarker in HF has been recently extensively studied, reported in the literature, and reviewed [4,5,7,9,82,83]. Baccouche et al. found a significant positive association between circulating Gal-3 and the risk of incident HF, based on a review of PUBMED-indexed peer-reviewed literature (9 studies). Adjusted meta-analysis presents a 32% higher HF risk in individuals with top-quartile log Gal-3 levels than in the bottom quartile [84]. Heart failure is commonly classified based on LV EF into the categories of HF with preserved (HFpEF, LV EF ≥ 50%), mildly reduced (HFmrEF), and reduced EF (HFrEF, LV EF < 40%) [1].

Alternative paradigms for HFpEF and HFrEF have been proposed due to a different pathophysiological mechanism. In HFpEF, systemic inflammation, microvascular endothelial dysfunction, cardiomyocyte hypertrophy, stiffening and eventually interstitial fibrosis are observed. In contrast, in HFrEF, myocardial damage and loss of viable cardiomyocytes predominate and play a major role in chamber dilation, elevated intracardiac pressures, and eccentric remodeling [85]

Subpopulations of HF patients vary in terms of characteristics and patterns of LV reverse remodeling. Kanaga et al. demonstrated that HFrEF patients have lower LVEF, increased LV volumes, a greater burden of focal and diffuse fibrosis, more right ventricular (RV) remodeling, lower left atrial (LA) EF, and higher LA volumes compared with HFpEF. HFrEF patients present eccentric LV remodeling expressed as ratio mass/volume, while in HFpEF, the concentric type was found. Cardiomyocyte stretch/stress was greater in HFrEF. However, serum Gal-3 was elevated in all HF patients compared with controls [86].

The correlation between Gal-3 overexpression and a bad prognosis for cardiac diseases has been proved in vitro and in vivo. When exposed to recombinant Gal-3, cardiac fibroblasts showed an increased collagen production, which was absent in Gal-3 knockdown, indicating a direct role of galectin in the fibrotic tissue generation during remodeling [87,88].

### 4.1. Symptoms, Exercise Capacity

Exercise intolerance in patients with chronic HF is one of the main symptoms and has a strong predictive value for prognosis. However, the relations between Gal-3 and exercise capacity in HF patients are ambiguous. Some studies found negative correlations between Gal-3 plasma or serum concentrations and peak VO_2_ in HF patients [89], but others did not [90,91]. In a recent study, Gal-3 serum concentration was associated with older age, female sex, and deeper impairment of renal function. Lower peak VO_2_ was associated with higher Gal-3 serum concentrations and more pronounced renal impairment [92]. In a recent study of 1515 participants, Gal-3 was inversely associated with exercise capacity. Patients with LV EF < 40%, previous myocardial infarction, AF, chronic lung disease, severe renal disease (estimated glomerular filtration rate <30 mL/min/m^2^), a history of cancer, and extreme values of Gal-3 (<1st percentile; >99th percentile) were excluded from that study [93].

Lack of exercise capacity improvement after cardiac rehabilitation can be related to higher inflammatory status. In a study by Fernandes-Silva et al., exercise capacity was not improved after a cardiac rehabilitation program in patients with Gal-3 serum concentration below the median of 6.2 (2.0) ng/mL [94]. Exercise capacity improvement, correlated with decreased Gal-3 and other cardiac biomarkers after training sessions, was revealed by Billebeau et al. It could be explained by the improvement of neurohormonal regulation after cardiac rehabilitation [95]. In a recent study of sedentary patients with cardiovascular risk factors, C-reactive protein, Gal-3, and insulin resistance significantly decreased after 8 weeks of exercise training [96].

### 4.2. Imaging Biomarkers

Echocardiography is the first-line diagnostic imaging modality to assess heart chambers geometry and function. However, its ability for tissue characterization is limited.

There are reports showing that serum Gal-3 concentration was significantly correlated with echocardiographic parameters for diastolic dysfunction, especially in patients with advanced HFpEF. Wu et al. published the results of a very interesting study in which they used three models of diastolic dysfunction: a human model of diastolic heart failure, a canine model of diastolic dysfunction, and in vitro cellular model of pressure overload. All patients included in the study had LVEF ≥ 50% and were divided into two groups: severe HFpEF (E/e′ ≥ 15) and mild HFpEF (E/e′ 8–15). Higher serum Gal-3 concentrations were found in patients with severe HFpEF than in mild HFpEF (19.4 ± 12.4 ng/mL vs. 6.8 ± 5.3 ng/L, respectively, *p* < 0.001). A significant correlation between Gal-3 and E/e′ was noted in patients with advanced HFpEF. In the animal model, cardiac Gal-3 increased significantly after pressure overload. Moreover, the expression of Gal-3 paralleled the severity of LV diastolic dysfunction. In the cellular model, cardiomyocytes produced and secreted more Gal-3 after mechanical stretch. The authors conclude that serum Gal-3 concentration may directly reflect changes in LV diastolic function or cardiac fibrosis and may serve as a sensitive marker to monitor the effect of treatment [97]. Other studies investigated the relationship between Gal-3 and LV function in patients with various clinical conditions [98,99]. The serum Gal-3 concentrations corresponded with echocardiographic indices (E/A, E/e′) and reflected the echocardiographic grades of LV diastolic dysfunction. In turn, a meta-analysis performed by Shi et al. showed that elevated serum Gal-3 concentrations were significantly related to E/e′ ratio and E-wave deceleration time (DT) but not to the E/A ratio, LA volume index, and LV mass [100].

There is no consensus in the literature on Gal-3’s relevance in LV geometry and function parameters. Lisowska et al. demonstrated that no correlation between the LV EF value and Gal-3 concentration was revealed in patients with myocardial infarction and stable coronary artery disease [101]. In the study consisting of 240 HFrEF patients, Lok et al. found a significant correlation between Gal-3 levels and changes in LV end-diastolic volume but no correlation between Gal-3 levels and LV end-diastolic volume value [102]. In patients with modest LV enlargement, the elevation of Gal-3 levels and LV remodeling was more prominent than in patients with larger ventricles at baseline, and those patients developed reverse remodeling more frequently. This observation suggests that Gal-3, a marker of inflammation and fibrosis, is especially elevated in HF patients with LV remodeling compared with patients without LV remodeling. Zaborska et al. found in symptomatic patients with chronic HFrEF that elevated Gal-3 concentrations were related to worsened RV function but not LV function. In the group with higher Gal-3 concentrations, an impairment in RV long-axis function, higher pulmonary artery systolic pressure, and increased right atrial pressure and, therefore, a lower index of RV-to-pulmonary circulation coupling, which is one of the most important noninvasive indexes in terms of RV failure assessment, were observed [103]. Of note, RV function has proven to be a prognostic factor in HFpEF and HFrEF. Similar observations were previously reported in AHF patients by Shah et al. [104]. Gal-3 levels were significantly associated with echocardiographic markers of LV filling and diastolic, not-systolic, function. Moreover, the authors noticed a striking relationship between indices of RV function and Gal-3 levels. The authors discussed elevated LV filling pressure and direct RV pathology due to fibrosis, remodeling, or hypertrophy as possible explanations.

Cardiac magnetic resonance (CMR) serves as a gold standard in imaging for tissue characterization. CMR allows for the determination of focal and diffuse fibrosis by late gadolinium enhancement (LGE) and post-contrast T1 time of the non-LGE myocardium. Recently Screever EM et al. demonstrated in a MI murine model and a well-characterized cohort of HF patients that diffused myocardial fibrosis on CMR was related to Gal-3 levels [105].

### 4.3. Heart Failure with Preserved Ejection Fraction

#### 4.3.1. Risk of Development

Gal-3 can be used to better characterize individuals at risk of HFpEF. Watson et al. performed a retrospective analysis of the STOP-HF study [106] of asymptomatic patients with risk factors for the future development of HFpEF [107]. Gal-3 was quantified at two time points prior to initial HFpEF diagnosis and was predictive of new onset HFpEF at the time point closest to the event. Another study evaluated the ability of four serum biomarkers (brain natriuretic peptide (BNP), Gal-3, N-terminal propeptide of procollagen type III (P3NP), and soluble ST2) to detect LV hypertrophy and diastolic dysfunction [108]. A total of 1705 subjects from the STANISLAS cohort were included in this analysis. The participants with LV hypertrophy or/and diastolic dysfunction had significantly higher Gal-3 serum concentrations than persons without these changes. Furthermore, Gal-3 appeared to be the most promising biomarker to detect preclinical LV diastolic dysfunction (C-index of Gal-3 0.64 vs. 0.62 for BNP, 0.55 for ST2, and 0.54 for P3NP). However, Lorenzo-Almorós et al. reported in their study that Gal -3 predicted symptomatic HF development in patients with chronic ischemic heart disease and type 2 diabetes mellitus but not in patients without DM [109].

#### 4.3.2. The Prognostic Value

Gal-3 may be an additional predictor of prognosis in HFpEF patients. The authors of the 2021 universal definition and classification of heart failure proposed four stages of HF: stage A—at risk of HF, stage B—pre-HF, stage C—symptomatic HF, and Stage D—advanced HF [110]. Mohebi et al. measured plasma biomarkers, including Gal-3, in patients undergoing coronary and/or peripheral angiography with or without intervention. The participants were stratified by HF stages. In the patients with stage A/B but not C/D, Gal-3 was independently associated with incident CV death or HF hospitalization (HR = 1.52, *p* = 0.03), [111]. Much information about the role of Gal-3 in the diagnosis and prognosis of HFpEF was provided by the results of the Diast-CHF study. The study included patients aged 50 to 85 years with ≥1 risk factor for HF or a history of HF. The subjects were observed for 10 years. In a head-to-head comparison between Gal-3 and NT-proBNP, Gal-3 ≥ 13.4 ng/mL outperformed NT-proBNP ≥ 220/660 pg/mL with an NRI of 0.22, z = 3.96, *p* < 0.001, for the diagnosis of HFpEF at baseline. During follow-up, patients without HFpEF at baseline and Gal-3 ≥ 13.4 ng/mL developed incident HFpEF significantly more often than patients with Gal-3 < 13.4 (*p* < 0.001). Gal-3 serum concentration ≥ 13.4 ng/mL was associated with a higher risk of all-cause mortality or a composite of cardiovascular hospitalization and death [112]. In addition, the above-mentioned meta-analysis by Shi et al. revealed that high serum Gal-3 concentrations were associated with a high risk of adverse outcomes (all-cause death, composite events: all-cause death and HF hospitalization or CV death and HF hospitalization) in patients with HFpEF [100].

### 4.4. Heart Failure with Reduced Ejection Fraction

Several early studies proved that Gal-3 in HF could be a prognostic marker for mortality and rehospitalization^.^ in short-term observation. Lok et al. proved that Gal-3 was associated with LV remodeling determined by serial echocardiography and predicted long-term mortality in patients with severe chronic HF [102]. The study was conducted on a group of 240 HF patients in New York Heart Association (NYHA) Class III and IV followed for 8.7 ± 1 years. Gal-3 levels were positively correlated with a change in LVEDV (*p* = 0.007). In addition, Gal-3 was a significant predictor of mortality after long-term follow-up (*p* = 0.001). However, Srivatsan et al. in their systemic overview concluded that the current weight of evidence did not suggest that Gal-3 was a predictor of all-cause mortality when factors such as renal failure, NT-pro BNP and LVEF were taken into consideration [113]. Doubts that the prognostic performance of Gal-3 in HFrEF is lower than that of other molecules, such as NT-proBNP or sST2, and influenced by renal function are still being raised by [4] Castiglione (2022). Recently, Cheng et al. performed a meta-analysis (pooled data included the results from 6440 patients from 12 studies) to evaluate the association between serum Gal-3 and all-cause and cardiovascular death in patients with chronic HF [8]. Higher serum Gal-3 was associated with a higher risk of all-cause (HR, 1.38; 95% CI, 1.14–1.67) and cardiovascular death (HR, 1.13; 95% CI, 1.02–1.25) in chronic HF patients. The authors of the PARADIGM-HF study aimed to answer the question of how much candidate biomarkers, with Gal-3 among them, improved risk prediction when added to comprehensive models, including routinely collected clinical and laboratory variables in HF. The study group consisted of 1559 patients in the NYHA II class. During a mean follow-up of 30.7 months, 300 patients experienced the primary outcome and 197 died. No meaningful improvement in the prediction of outcomes over what is provided by clinical, routine laboratory, and natriuretic peptide variables has been found [114]. However, there is also a different view on the use of Gal-3 in a risk and prognosis assessment in HF patients presented by Shah et al. as a tool for patient-tailored management [83]. The authors stressed the essential role of Gal-3 in guiding physicians to identify HF patients at risk of decompensation, readmission, and death, and the importance of timing since Gal-3 expression is maximal at peak fibrosis and virtually absent after recovery [83].

#### 4.4.1. Cardiac Resynchronization Therapy

Cardiac resynchronization therapy (CRT) has become a standard, valuable mode of treatment for HFrEF patients who remain symptomatic despite optimal medical therapy. However, this is an interventional therapy with a reported 60–80% rate of response and the risk/benefit ratio must be taken into consideration during patients’ qualification for this procedure. Excessive cardiac fibrosis and the presence of a myocardial scar are the likely cause of worse prognosis in patients with elevated Gal-3 concentration. The role of Gal-3 and other biomarkers of myocardial fibrosis in HF patients receiving CRT has been investigated in several studies and the results obtained are not fully consistent. In the CARE-HF trial substudy, serial changes of collagen turnover biomarkers and CRT effects on these markers were examined. The study showed that CRT did not lead to significant changes in Gal-3 concentration during 18 months of follow-up [115]. The authors concluded that extracellular cardiac remodeling may not be involved in the CRT’s beneficial effect. However, the baseline serum Gal-3 concentration was associated with death or hospitalization for HF worsening at 18 months. This association was independent of NT-pro BNP concentration, although it became insignificant after adjusting for renal function, expressed as eGFR value [115]. In the MADIT-CRT trial, in which 654 patients with mild HF symptoms were randomized to CRT or implantable cardioverter defibrillator, an elevated Gal-3 concentration was a significant and independent predictor of nonfatal HF event or death [116]. Andre et al. confirmed the role of Gal-3 in predicting response to CRT and long-term outcomes defined as death and hospitalization for MACE composite of hospitalization for HF, cardiogenic shock, and sustained ventricular tachycardia in patients with typical left bundle branch block (LBBB) [48]. Gal-3 serum concentration equal to or exceeding 22 ng/mL predicted survival after CRT implantation at 48-month follow-up [48]. Recently, Zaborska et al. showed that baseline log Gal-3 concentration and RV function expressed as tricuspid annular plane systolic excursion were independent predictors of 5-year all-cause mortality, with HR 2.96 (*p*  =  0.037) and HR 0.88 (*p*  =  0.023) respectively, in HFrEF patients following CRT implantation [117]. In the studied patient cohort, the median serum Gal-3 concentration was 13.4 ng/mL (IQR 11.05, 17.15). The analysis of subgroups defined by Gal-3 concentration and CRT response showed that patients with high baseline Gal-3 concentrations and a lack of response to CRT had a significantly lower probability of survival [117].

#### 4.4.2. Right Ventricular Dysfunction

Right ventricular dysfunction in HF patients results from various pathological mechanisms, including RV injury, remodeling, fibrosis, and pulmonary hypertension, and leads to a worsening of the prognosis. Recently, potential mechanisms underlying the relationship between Gal-3 and RV function have been demonstrated. An established RV fibrosis was found to be characterized by marked expression of Gal-3 and enhanced numbers of proliferating RV fibroblasts in patients with pulmonary hypertension and experimental animal models [118]. Moreover, He et al. found that Gal-3-mediated pulmonary artery hypertension through NADPH oxidase 4 and NADPH oxidase 4-derived oxidative stress led to RV remodeling [87]. RV dysfunction and pulmonary hypertension are well-known complications in patients with congenital heart disease. In this population, Shen et al. reported a facilitating role of Gal-3 in pulmonary artery remodeling and progression of pulmonary artery hypertension [119]. Demonstrating the link between RV function, pulmonary hypertension and Gal-3 may result in a potential therapeutic approach. Hao et al. showed that Gal-3 inhibition ameliorated hypoxia-induced pulmonary artery hypertension and reduced the inflammatory response in an animal model [120].

## 5. Heart Failure with Atrial Fibrillation

Atrial fibrillation often coexists with HF, and growing evidence suggests that myocardial fibrosis is a contributing factor for both AF and HF development [121,122]. Recent years have brought much information about its complex and multifactorial nature. AF is the most common cardiac arrhythmia and one of the leading risk factors for morbidity and mortality [123]. AF is a major mechanism of LV diastolic dysfunction as it results in the reduction of LV filling due to the absence of atrial systole and is present in more than half of patients with HFpEF [124]. Moreover, a stiff left ventricle also causes increased atrial pressure and stretch resulting in AF.

Atrial interstitial fibrosis is the hallmark of LA remodeling and perpetuation of arrhythmia [121]. Atrial fibrosis may develop as part of AF-related structural remodeling and as a consequence of other cardiovascular diseases that result in oxidative stress, inflammation, atrial overload, and stretch [125]. Conditions associated with atrial fibrosis include aging, HA, valvular heart disease, coronary artery disease, and HF, causing broadly similar histological changes in the atrial myocardium. In recent years, the term atrial cardiomyopathy has been introduced, which is defined as any complex structural, architectural, contractile, or electrophysiological changes affecting the atria with the potential to produce clinically relevant manifestations [126].

Fibrotic tissue formation is a complex multifactorial process involving many interactions of neurohumoral and cellular factors [127]. Gal-3 is a soluble beta-galactoside-binding lectin that mediates cardiac fibroblast proliferation resulting in fibrosis. The results of the studies demonstrated a higher level of Gal-3 in patients with AF [128] and a correlation of serum concentration of Gal-3 with atrial remodeling [128,129]. The study with the use of delayed-enhancement magnetic resonance imaging (DE-MRI) showed that Gal-3 and LA volume index were independently correlated with the range of LA fibrosis in patients with paroxysmal AF with preserved LV function [130]. The observational study by Hernandez-Romero et al. [131] demonstrated an association between Gal-3 serum concentration and interstitial fibrosis assessed in LA appendage tissue samples obtained during cardiac surgery in patients with aortic valve or ischemic heart diseases. Previous cardiac disease, NYHA scale, and high Gal-3 were independent predictors of LA fibrosis. Moreover, the study demonstrated that atrial tissue fibrosis was an independent factor for postoperative AF occurrence.

The role of Gal-3 as a prognostic marker was studied in AF and HF populations with reduced, mildly reduced, and preserved EF. It was revealed that an increased level of Gal-3 was associated with an increased risk of mortality in patients with AF [132]. Nonetheless, the study did not demonstrate that elevated Gal-3 serum concentration was associated with prevalent AF in the HF population. The opposite result was shown in the prospective study by Aksan et al. in HF patients undergoing CRT [133]. The study revealed that Gal-3 serum concentration was significantly higher in patients with atrial high-rate (AHR) episodes, which were related to AF than in those without AHR episodes. Moreover, Gal-3 concentration was a predictor for the development of AHR episodes in the long-term follow-up in patients with HF undergoing CRT.

There is still a need to better understand the role of the biomarkers, including Gal-3, in the pathomechanism of HF with coexisting AF. An assessment of the extent of fibrosis may be useful to determine the prognosis in such patients.

## 6. Gal-3 as a Therapeutic Target for Heart Failure

New treatment methods for HF and conditions predisposing to its occurrence are constantly searched for. The data from preclinical studies based on deletions of the Gal-3 encoding gene showing the role of Gal-3 in cardiovascular diseases gave an impetus to the development of Gal-3 targeted therapy [134,135,136]. Therefore, new compounds have been tested in several studies (Table 1).

There is potential for Gal-3 as a therapeutic target in fibrosis and, therefore, in HF. It is promising as a concept. However, no compound is ready to be used in a clinical setting in HF. Furthermore, at present, only TDI 139, a selective Gal-3 inhibitor, was investigated in phase Ia/Ib trials for safety, tolerability, pharmacokinetics, and biomarkers and in phase IIb trials in idiopathic pulmonary fibrosis patients (Table 1). Data from in vitro studies and from animal models are conflicting. The main problems are the selectivity of Gal-3 inhibitors, the timing of disease progression in various models, and challenges related to dosing, delivery, and off-target effects of pharmacologic inhibitors. Most pharmacological approaches have been built on the high-resolution crystal structure of the CRD of Gal-3 in a complex with lactose. Although it is the main component required to drive the Gal-3 biological functions, carbohydrate–protein interactions are weak [28]. The rational design of more potent and selective Gal-3 inhibitors is based on structural and biophysical studies, which led to the knowledge of the nature and thermodynamics of Gal-3 ligand interaction. To enhance the potency, a skeleton of natural di- or monosaccharides has been decorated with various aromatic substituents to establish strong interaction within the binding site [28].

Inconsistencies between studies using different models of the HF reflect context-dependent actions of Gal-3 [146]. In our opinion, the bottleneck is the fact that a single animal model cannot recapitulate the remarkable pathophysiological heterogeneity of human HF.

There are scarce data regarding ongoing clinical trials testing galectin-3 inhibitors to treat heart failure. Currently, no clinical trial regarding galectin inhibitors and heart failure is registered on ClinicalTrials.gov.

Only a few clinical studies have presented data regarding the relationship between serum Gal-3 concentrations and the response to specific pharmacological interventions for HFpEF. The detrimental effects of aldosterone may, in part, be conferred via Gal-3, hence the suggestion that aldosterone blockers may have a favorable effect in patients with high serum Gal-3 concentrations. Symptomatic HF patients, NYHA class II or III, and peak VO2 ≤ 25 mL/kg/min in cardiopulmonary test, with preserved LV systolic function, LVEF ≥ 50% at rest but overt LV diastolic dysfunction (echocardiographic evidence of grade ≥ I or present AF), were included into the Aldo-DHF study. The patients were randomized to spironolactone 25 mg once daily or placebo. Serum Gal-3 concentrations were not significantly different between the groups during 12 months of follow-up [147]. The population of the HOMAGE randomized trial was at increased risk of developing HF. It consisted of 527 participants with, or at high risk of, coronary disease and raised plasma B-type natriuretic peptides. Gal-3 did not identify greater reductions in serum concentrations of collagen biomarkers in response to 50 mg spironolactone daily [148]. In the TOPCAT trial spironolactone appeared to alter the determinants of extracellular matrix remodeling in an anti-fibrotic fashion in patients with diabetes, reflected by changes in hs-TnT and TIMP-1 levels but not in Gal-3 over time [149]. Interestingly, a study in a group of patients with HFrEF revealed contrary results. The cross-sectional descriptive study was conducted on 122 patients, nonusers of spironolactone. After 12 weeks of treatment with spironolactone, the Gal-3 concentration decreased from 54.82 ± 26.06 to 44.20 ± 24.36 (*p* < 0.05). The 50 mg once-daily dose of spironolactone significantly improved Gal-3 concentrations compared with the 25 mg once-daily group, at 17.11 ± 20.81 (*p* < 0.05) (reduced by 29.05%) and 3.46 ± 6.81 ng/mL (*p* < 0.05) (reduced by 6.87%), respectively [150].

The above-mentioned mechanisms do not exhaust the potential routes of Gal-3 targeted intervention. Enhanced β-adrenoceptor activity is a determinant of both circulating concentration and cardiac expression of Gal-3. Pharmacological or transgenic activation of β-adrenoceptors leads to increased blood levels of Gal-3 and upregulated cardiac Gal-3 expression, an effect that can be reversed with the use of β-adrenoceptor antagonists [151]. In a recent experimental study, Zhao et al. revealed that stimulation of cardiac β-adrenoceptors activated the Mst1/Hippo pathway, leading to YAP hyper-phosphorylation with enhanced expression of Gal-3 and BIM. This signaling pathway would have therapeutic potential [152].

The mechanisms underlying the development of HF and its further dynamics, progression, exacerbations, and various clinical presentation are numerous and complex. However, fibrosis is the hallmark of HF, and even in this field remain problems to be solved. We encounter different types of fibrosis as a focal myocardial scar or diffuse or interstitial fibrosis. There are some data that Gal-3 is related to diffuse fibrosis. The role of diffuse fibrosis and its usefulness regarding prognosis might differ between different HF etiologies [105]. The type of fibrosis determines whether this process is reversible, at least to some point. Timing of assessment and decision of potential therapeutic intervention is also crucial since we could encounter ongoing or completed fibrosis and remodeling. The data on longitudinal changes in Gal-3 levels are scarce. The relatively new field is the search for factors responsible for Gal-3 gene expression, especially among noncoding RNAs.

An understanding of the Gal-3 biology and HF underlying mechanisms might give a chance to bring a new anti-fibrotic treatment to a selected group of patients.

## 7. Unsolved Issues and Perspectives

One of the still-burning basal questions is Gal-3 concentration normal reference ranges. It remains open and mainly depends on the tested population. The data on values of Gal-3 in healthy subjects are relatively scarce and results are also sometimes conflicting. Agnello et al. recruited 706 blood donors and found that the median Gal-3 concentration was 14.3 (IQR 11.9–16.7) ng/mL with 97.5th percentile URL 26.1 ng/mL, and it was related to age [153]. Mueller et al. also examined blood donors and concluded that the 97.5th percentile URL for Gal-3 was 16 ng/mL in males and 17 ng/mL in females [154]. An improvement in the detection methods of Gal-3 is needed with respect to the sensitivity, accuracy, and consensus between different laboratories.

Gal-3 is a potent marker of fibrosis found in a wide range of tissues and, therefore, is involved in many relevant human diseases: cardiac, pulmonary, liver, and kidney [145]. In addition, it plays a regulatory role in inflammation and cancer [155]. Gal-3 is even called one molecule for an Alphabet of Diseases, from A to Z [156]. According to our knowledge, up till now, no algorithms of Gal-3 level assessment that allow for distinguishing between cardiac and noncardiac causes have been developed. Cutoff values for risk assessment mainly depend on the tested population. Therefore, imaging methods should be included in the differential diagnosis at this stage. However, Gal-3 nonspecificity could be also an advantage since advanced HF is a complex clinical syndrome with multiorgan involvement, and a condition of other target organs, such as the liver, kidney, and lungs, must be taken into account.

The perspectives are mainly associated with Gal-3 inhibitors, as promising therapeutic agents. However, we must note how much still needs to be clarified in terms of clinical effectiveness, pharmacokinetics, and potential side effects.

One of the main molecular pathomechanisms of cardiovascular disease (CVD), and especially heart failure, is an imbalance in the expression of noncoding RNAs (ncRNAs) [157]. Noncoding RNAs are a class of transcripts that do not encode proteins. They include microRNAs (miRNAs), long noncoding RNAs (lncRNAs), and circular RNAs (circRNAs). They have important biological functions, such as regulating transcription and translation, as well as interacting with DNA, RNA, and proteins. Increased or decreased expression of specific ncRNAs leads to the development of CVD [158,159]. It appears that restoring the balance of ncRNA expression may represent a new and effective strategy for treating CVD. Circulating ncRNAs also show great potential as noninvasive biomarkers of CVD, alone or in combination with classical biomarkers. Among circulating ncRNAs, plasma and serum miRNAs are considered potential biomarkers for many CVDs and are widely used in diagnosis, prognosis, and assessment of treatment response [160]. Other circulating ncRNAs, including lncRNAs and circRNAs, are also promising biomarkers of CVD [127]. The biological functions of ncRNAs that are currently known are just the tip of the iceberg. Understanding the underlying pathophysiological mechanisms and further identifying new potential treatment targets may improve the poor prognosis of patients with chronic HF. An in-depth understanding of the role of ncRNAs in CVD requires further research that will lay the foundation for the development of new disease treatment programs.

## 8. Conclusions

Heart failure is a clinical syndrome with high morbidity and mortality, and its prevalence is still rapidly increasing. The growing interest in biomarkers useful in diagnostics and risk stratification has been reflected in the literature. Gal-3 is a unique, fascinating lectin with respect to structure and function. Gal-3 is an important factor in the pathophysiology of HF, mainly in view of its role in cardiac fibrosis, inflammation, and cardiac ventricular remodeling. Fibrosis is a hallmark of cardiac remodeling and the development of HF and AF. Numerous studies have shown an association between echocardiographic and CMR biomarkers in HF and Gal-3 serum concentration. This could pave the way for the combined use of imaging and biochemical biomarkers to improve diagnostic and prognostic accuracy.

Recently, a positive association between Gal-3 concentration and the incidence of HF has been proved. Gal-3 is a valuable prognostic marker in acute and chronic HF for both HpEF and HrEF. Higher Gal-3 concentration indicates a higher risk of all-cause and cardiovascular mortality and a higher risk of complications. Whether Gal-3 prognostic value is independent of renal and hepatic function parameters and incremental of other well-known biomarkers, such as natriuretic peptides, remains debatable. However, based on the growing knowledge of the molecular properties of Gal-3 and its complex mechanism of action, as well as advancing knowledge of the pathophysiology of HF and the therapeutic approach, there is a space for tailoring medical procedures depending on the characteristics of the patient population and the time point of the developing pathology. This direction requires further investigation. Furthermore, understanding the Gal-3 biology, especially on the genetic level, and HF pathology enables promising research on Gal-3 as a therapeutic target.

Although the current results indicate potential clinical applications in humans, the clinical utility of treatment strategies targeting elevated Gal-3 concentrations remains to be explored.

## Figures and Tables

**Table 1:** Potential therapeutic Inhibitors of Galectin-3.

Compound	Model	Function	Comment	References
**Monosaccharides** N-acetyllactosamine	HF-susceptible REN2 rats	Prevention of LV dysfunction.	Rapidly absorbed and metabolized.	[137]
**Neoglycoproteins** LacDiNAc	Human Gal-3	Anti-fibrosis action through serving as a ligand with a high affinity High selectivity for Gal-3.	Clinical context needs to be assessed.	[138]
**Galactomannans** Modified Citrus Pectins (MCPs) Modified Rhubarb Pectin (EMRP) Selectivity unknown	Numerous cell and animal models	Inhibition of *LGALS3* gene expression reduction of expression of proinflammatory cytokines (IL-1β, IL-18, TNF-α) involved in the pathogenesis of HF. Inhibition of heart inflammation. Reduction of atherosclerosis and decrease of the degree of myocardial damage. Attenuation of cardiac fibrosis and reduction of collagen deposition.	No MCPs specificity was shown in in vivo studies. Natural rhubarb pectin has a superior inhibitory capacity over established pectins.	[139,140,141,142,143]
**Thiodigalactosides** TD-139	Healthy subjects and patients with idiopathic pulmonary fibrosis	Modulation of CRD of Gal-3 Treatment of idiopathic pulmonary fibrosis. Suppression of Gal-3 expression on bronchoalveolar lavage macrophages.	TD-139 approved by FDA.	[144,145]

## Abbreviations

Gal-3—galectin-3; CRD—carbohydrate recognition domain; NF-κB—nuclear factor κB; MCL-1—myeloid cell leukemia sequence 1 protein; KLF-3—Kruppler-like factor 3; RUNX2—runt-related transcription factor 2; ECM—extracellular matrix; COL1A1—extracellular type 1 collagen α-1 chain (an extracellular fibrosis marker); MMP—matrix metalloproteinase; TIMP—tissue inhibitor metalloproteinase; α-SMA—cytoskeletal protein α-smooth muscle actin (an intracellular fibrosis marker); PTEN—phosphatase and tensin homolog; PTK2—protein tyrosine kinase 2; HF—heart failure; EF—ejection fraction; HFrEF—heart failure with reduced ejection fraction; HFpEF—heart failure with preserved ejection fraction; LV—left ventricular; RV—right ventricular; LA—left atrium; AF—atrial fibrillation; CRT—cardiac resynchronization therapy; CVD—cardiovascular disease; DM—diabetes mellitus.

## References

[1] McDonagh T.A., Metra M., Adamo M., Gardner R.S., Baumbach A., Bohm M., Burri H., Butler J., Celutkiene J., Chioncel O. (2021). 2021 ESC Guidelines for the diagnosis and treatment of acute and chronic heart failure. Eur. Heart J..

[2] Savarese G., Becher P.M., Lund L.H., Seferovic P., Rosano G.M.C., Coats A.J.S. (2023). Global burden of heart failure: A comprehensive and updated review of epidemiology. Cardiovasc. Res..

[3] Heidenreich P.A., Bozkurt B., Aguilar D., Allen L.A., Byun J.J., Colvin M.M., Deswal A., Drazner M.H., Dunlay S.M., Evers L.R. (2022). 2022 AHA/ACC/HFSA Guideline for the Management of Heart Failure: A Report of the American College of Cardiology/American Heart Association Joint Committee on Clinical Practice Guidelines. Circulation.

[4] Castiglione V., Aimo A., Vergaro G., Saccaro L., Passino C., Emdin M. (2022). Biomarkers for the diagnosis and management of heart failure. Heart Fail. Rev..

[5] Blanda V., Bracale U.M., Di Taranto M.D., Fortunato G. (2020). Galectin-3 in Cardiovascular Diseases. Int. J. Mol. Sci..

[6] Suthahar N., Meijers W.C., Silljé H.H.W., Ho J.E., Liu F.T., de Boer R.A. (2018). Galectin-3 Activation and Inhibition in Heart Failure and Cardiovascular Disease: An Update. Theranostics.

[7] Bosnjak I., Selthofer-Relatic K., Vcev A. (2015). Prognostic value of galectin-3 in patients with heart failure. Dis. Markers.

[8] Cheng Z., Cai K., Xu C., Zhan Q., Xu X., Xu D., Zeng Q. (2022). Prognostic Value of Serum Galectin-3 in Chronic Heart Failure: A Meta-Analysis. Front. Cardiovasc. Med..

[9] Sun R.R., Lu L., Liu M., Cao Y., Li X.C., Liu H., Wang J., Zhang P.Y. (2014). Biomarkers and heart disease. Eur. Rev. Med. Pharmacol. Sci..

[10] Chow S.L., Maisel A.S., Anand I., Bozkurt B., de Boer R.A., Felker G.M., Fonarow G.C., Greenberg B., Januzzi J.L., Kiernan M.S. (2017). Role of Biomarkers for the Prevention, Assessment, and Management of Heart Failure: A Scientific Statement From the American Heart Association. Circulation.

[11] Meijers W.C., Bayes-Genis A., Mebazaa A., Bauersachs J., Cleland J.G.F., Coats A.J.S., Januzzi J.L., Maisel A.S., McDonald K., Mueller T. (2021). Circulating heart failure biomarkers beyond natriuretic peptides: Review from the Biomarker Study Group of the Heart Failure Association (HFA), European Society of Cardiology (ESC). Eur. J. Heart Fail..

[12] Liu F.T., Patterson R.J., Wang J.L. (2002). Intracellular functions of galectins. Biochim. Biophys. Acta.

[13] Yang R.Y., Rabinovich G.A., Liu F.T. (2008). Galectins: Structure, function and therapeutic potential. Expert. Rev. Mol. Med..

[14] Chen H.Y., Weng I.C., Hong M.H., Liu F.T. (2014). Galectins as bacterial sensors in the host innate response. Curr. Opin. Microbiol..

[15] Cooper D.N. (2002). Galectinomics: Finding themes in complexity. Biochim. Biophys. Acta.

[16] Rabinovich G.A., Toscano M.A. (2009). Turning ‘sweet’ on immunity: Galectin-glycan interactions in immune tolerance and inflammation. Nat. Rev. Immunol..

[17] Nabi I.R., Shankar J., Dennis J.W. (2015). The galectin lattice at a glance. J. Cell Sci..

[18] Johannes L., Jacob R., Leffler H. (2018). Galectins at a glance. J. Cell Sci..

[19] Sygitowicz G., Maciejak-Jastrzebska A., Sitkiewicz D. (2021). The Diagnostic and Therapeutic Potential of Galectin-3 in Cardiovascular Diseases. Biomolecules.

[20] Kaminker J.D., Timoshenko A.V. (2021). Expression, Regulation, and Functions of the Galectin-16 Gene in Human Cells and Tissues. Biomolecules.

[21] Barboni E.A., Bawumia S., Henrick K., Hughes R.C. (2000). Molecular modeling and mutagenesis studies of the N-terminal domains of galectin-3: Evidence for participation with the C-terminal carbohydrate recognition domain in oligosaccharide binding. Glycobiology.

[22] Dumic J., Dabelic S., Flögel M. (2006). Galectin-3: An open-ended story. Biochim. Biophys. Acta.

[23] Raimond J., Rouleux F., Monsigny M., Legrand A. (1995). The second intron of the human galectin-3 gene has a strong promoter activity down-regulated by p53. FEBS Lett..

[24] Duneau M., Boyer-Guittaut M., Gonzalez P., Charpentier S., Normand T., Dubois M., Raimond J., Legrand A. (2005). Galig, a novel cell death gene that encodes a mitochondrial protein promoting cytochrome c release. Exp. Cell Res..

[25] Knights A.J., Yik J.J., Mat Jusoh H., Norton L.J., Funnell A.P., Pearson R.C., Bell-Anderson K.S., Crossley M., Quinlan K.G. (2016). Krüppel-like Factor 3 (KLF3/BKLF) Is Required for Widespread Repression of the Inflammatory Modulator Galectin-3 (Lgals3). J. Biol. Chem..

[26] Balan V., Nangia-Makker P., Jung Y.S., Wang Y., Raz A. (2010). Galectin-3: A novel substrate for c-Abl kinase. Biochim. Biophys. Acta.

[27] Li X., Ma Q., Wang J., Liu X., Yang Y., Zhao H., Wang Y., Jin Y., Zeng J., Li J. (2010). c-Abl and Arg tyrosine kinases regulate lysosomal degradation of the oncoprotein Galectin-3. Cell Death Differ..

[28] Bouffette S., Botez I., De Ceuninck F. (2023). Targeting galectin-3 in inflammatory and fibrotic diseases. Trends Pharmacol. Sci..

[29] Stock M., Schäfer H., Stricker S., Gross G., Mundlos S., Otto F. (2003). Expression of galectin-3 in skeletal tissues is controlled by Runx2. J. Biol. Chem..

[30] Menon R.P., Hughes R.C. (1999). Determinants in the N-terminal domains of galectin-3 for secretion by a novel pathway circumventing the endoplasmic reticulum-Golgi complex. Eur. J. Biochem..

[31] Sato S., Ouellet M., St-Pierre C., Tremblay M.J. (2012). Glycans, galectins, and HIV-1 infection. Ann. N. Y. Acad. Sci..

[32] Nielsen C.T., Østergaard O., Rasmussen N.S., Jacobsen S., Heegaard N.H.H. (2017). A review of studies of the proteomes of circulating microparticles: Key roles for galectin-3-binding protein-expressing microparticles in vascular diseases and systemic lupus erythematosus. Clin. Proteom..

[33] Noguchi K., Tomita H., Kanayama T., Niwa A., Hatano Y., Hoshi M., Sugie S., Okada H., Niwa M., Hara A. (2019). Time-course analysis of cardiac and serum galectin-3 in viral myocarditis after an encephalomyocarditis virus inoculation. PLoS ONE.

[34] Kobayashi K., Niwa M., Hoshi M., Saito K., Hisamatsu K., Hatano Y., Tomita H., Miyazaki T., Hara A. (2015). Early microlesion of viral encephalitis confirmed by galectin-3 expression after a virus inoculation. Neurosci. Lett..

[35] De Oliveira F.L., Gatto M., Bassi N., Luisetto R., Ghirardello A., Punzi L., Doria A. (2015). Galectin-3 in autoimmunity and autoimmune diseases. Exp. Biol. Med..

[36] Dhirapong A., Lleo A., Leung P., Gershwin M.E., Liu F.T. (2009). The immunological potential of galectin-1 and -3. Autoimmun. Rev..

[37] Shin T. (2013). The pleiotropic effects of galectin-3 in neuroinflammation: A review. Acta Histochem..

[38] Saccon F., Gatto M., Ghirardello A., Iaccarino L., Punzi L., Doria A. (2017). Role of galectin-3 in autoimmune and non-autoimmune nephropathies. Autoimmun. Rev..

[39] Stitt A.W., He C., Vlassara H. (1999). Characterization of the advanced glycation end-product receptor complex in human vascular endothelial cells. Biochem. Biophys. Res. Commun..

[40] Inohara H., Raz A. (1995). Functional evidence that cell surface galectin-3 mediates homotypic cell adhesion. Cancer Res..

[41] Ochieng J., Leite-Browning M.L., Warfield P. (1998). Regulation of cellular adhesion to extracellular matrix proteins by galectin-3. Biochem. Biophys. Res. Commun..

[42] Vlassara H., Li Y.M., Imani F., Wojciechowicz D., Yang Z., Liu F.T., Cerami A. (1995). Identification of galectin-3 as a high-affinity binding protein for advanced glycation end products (AGE): A new member of the AGE-receptor complex. Mol. Med..

[43] Dagher S.F., Wang J.L., Patterson R.J. (1995). Identification of galectin-3 as a factor in pre-mRNA splicing. Proc. Natl. Acad. Sci. USA.

[44] Kim H.R., Lin H.M., Biliran H., Raz A. (1999). Cell cycle arrest and inhibition of anoikis by galectin-3 in human breast epithelial cells. Cancer Res..

[45] Yang R.Y., Hsu D.K., Liu F.T. (1996). Expression of galectin-3 modulates T-cell growth and apoptosis. Proc. Natl. Acad. Sci. USA.

[46] Akahani S., Nangia-Makker P., Inohara H., Kim H.R., Raz A. (1997). Galectin-3: A novel antiapoptotic molecule with a functional BH1 (NWGR) domain of Bcl-2 family. Cancer Res..

[47] Clementy N., Garcia B., André C., Bisson A., Benhenda N., Pierre B., Bernard A., Fauchier L., Piver E., Babuty D. (2018). Galectin-3 level predicts response to ablation and outcomes in patients with persistent atrial fibrillation and systolic heart failure. PLoS ONE.

[48] Andre C., Piver E., Perault R., Bisson A., Pucheux J., Vermes E., Pierre B., Fauchier L., Babuty D., Clementy N. (2018). Galectin-3 predicts response and outcomes after cardiac resynchronization therapy. J. Transl. Med..

[49] Zuern C.S., Floss N., Mueller I.I., Eick C., Duckheim M., Patzelt J., Gawaz M., May A.E., Mueller K.A.L. (2018). Galectin-3 is associated with left ventricular reverse remodeling and outcome after percutaneous mitral valve repair. Int. J. Cardiol..

[50] Asleh R., Enriquez-Sarano M., Jaffe A.S., Manemann S.M., Weston S.A., Jiang R., Roger V.L. (2019). Galectin-3 Levels and Outcomes After Myocardial Infarction: A Population-Based Study. J. Am. Coll. Cardiol..

[51] Cui Y., Qi X., Huang A., Li J., Hou W., Liu K. (2018). Differential and Predictive Value of Galectin-3 and Soluble Suppression of Tumorigenicity-2 (sST2) in Heart Failure with Preserved Ejection Fraction. Med. Sci. Monit..

[52] Dupuy A.M., Kuster N., Curinier C., Huet F., Plawecki M., Solecki K., Roubille F., Cristol J.P. (2019). Exploring collagen remodeling and regulation as prognosis biomarkers in stable heart failure. Clin. Chim. Acta.

[53] Ghorbani A., Bhambhani V., Christenson R.H., Meijers W.C., de Boer R.A., Levy D., Larson M.G., Ho J.E. (2018). Longitudinal Change in Galectin-3 and Incident Cardiovascular Outcomes. J. Am. Coll. Cardiol..

[54] Tan K.C.B., Cheung C.L., Lee A.C.H., Lam J.K.Y., Wong Y., Shiu S.W.M. (2018). Galectin-3 is independently associated with progression of nephropathy in type 2 diabetes mellitus. Diabetologia.

[55] Alam M.L., Katz R., Bellovich K.A., Bhat Z.Y., Brosius F.C., de Boer I.H., Gadegbeku C.A., Gipson D.S., Hawkins J.J., Himmelfarb J. (2019). Soluble ST2 and Galectin-3 and Progression of CKD. Kidney Int. Rep..

[56] Savoj J., Becerra B., Kim J.K., Fusaro M., Gallieni M., Lombardo D., Lau W.L. (2019). Utility of Cardiac Biomarkers in the Setting of Kidney Disease. Nephron.

[57] Chen S.C., Kuo P.L. (2016). The Role of Galectin-3 in the Kidneys. Int. J. Mol. Sci..

[58] Gopal D.M., Ayalon N., Wang Y.C., Siwik D., Sverdlov A., Donohue C., Perez A., Downing J., Apovian C., Silva V. (2019). Galectin-3 Is Associated With Stage B Metabolic Heart Disease and Pulmonary Hypertension in Young Obese Patients. J. Am. Heart Assoc..

[59] Siew J.J., Chen H.M., Chen H.Y., Chen H.L., Chen C.M., Soong B.W., Wu Y.R., Chang C.P., Chan Y.C., Lin C.H. (2019). Galectin-3 is required for the microglia-mediated brain inflammation in a model of Huntington’s disease. Nat. Commun..

[60] Ashraf G.M., Baeesa S.S. (2018). Investigation of Gal-3 Expression Pattern in Serum and Cerebrospinal Fluid of Patients Suffering From Neurodegenerative Disorders. Front. Neurosci..

[61] Wang L., Guo X.L. (2016). Molecular regulation of galectin-3 expression and therapeutic implication in cancer progression. Biomed. Pharmacother..

[62] Nangia-Makker P., Hogan V., Raz A. (2018). Galectin-3 and cancer stemness. Glycobiology.

[63] Wang C., Zhou X., Ma L., Zhuang Y., Wei Y., Zhang L., Jin S., Liang W., Shen X., Li C. (2019). Galectin-3 may serve as a marker for poor prognosis in colorectal cancer: A meta-analysis. Pathol. Res. Pract..

[64] Fermann G.J., Lindsell C.J., Storrow A.B., Hart K., Sperling M., Roll S., Weintraub N.L., Miller K.F., Maron D.J., Naftilan A.J. (2012). Galectin 3 complements BNP in risk stratification in acute heart failure. Biomarkers.

[65] Grandin E.W., Jarolim P., Murphy S.A., Ritterova L., Cannon C.P., Braunwald E., Morrow D.A. (2012). Galectin-3 and the development of heart failure after acute coronary syndrome: Pilot experience from PROVE IT-TIMI 22. Clin. Chem..

[66] González G.E., Cassaglia P., Noli Truant S., Fernández M.M., Wilensky L., Volberg V., Malchiodi E.L., Morales C., Gelpi R.J. (2014). Galectin-3 is essential for early wound healing and ventricular remodeling after myocardial infarction in mice. Int. J. Cardiol..

[67] Al-Salam S., Hashmi S., Jagadeesh G.S., Tariq S. (2020). Galectin-3: A Cardiomyocyte Antiapoptotic Mediator at 24-Hour Post Myocardial Infarction. Cell. Physiol. Biochem..

[68] Cassaglia P., Penas F., Betazza C., Fontana Estevez F., Miksztowicz V., Martínez Naya N., Llamosas M.C., Noli Truant S., Wilensky L., Volberg V. (2020). Genetic Deletion of Galectin-3 Alters the Temporal Evolution of Macrophage Infiltration and Healing Affecting the Cardiac Remodeling and Function after Myocardial Infarction in Mice. Am. J. Pathol..

[69] Sanchez-Mas J., Lax A., Asensio-Lopez M.C., Fernandez-Del Palacio M.J., Caballero L., Garrido I.P., Pastor F., Januzzi J.L., Pascual-Figal D.A. (2014). Galectin-3 expression in cardiac remodeling after myocardial infarction. Int. J. Cardiol..

[70] Di Tano G., Caretta G., De Maria R., Parolini M., Bassi L., Testa S., Pirelli S. (2017). Galectin-3 predicts left ventricular remodelling after anterior-wall myocardial infarction treated by primary percutaneous coronary intervention. Heart.

[71] Di Tano G., Caretta G., De Maria R., Bettari L., Parolini M., Testa S., Pirelli S. (2018). Galectin-3 and outcomes after anterior-wall myocardial infarction treated by primary percutaneous coronary intervention. Biomark. Med..

[72] Tymińska A., Kapłon-Cieślicka A., Ozierański K., Budnik M., Wancerz A., Sypień P., Peller M., Maksym J., Balsam P., Opolski G. (2019). Association of galectin-3 and soluble ST2 with in-hospital and 1-year outcomes in patients with ST-segment elevation myocardial infarction treated with primary percutaneous coronary intervention. Pol. Arch. Intern. Med..

[73] Köktürk U., Püşüroğlu H., Somuncu M.U., Akgül Ö., Uygur B., Özyılmaz S., Işıksaçan N., Sürgit Ö., Yıldırım A. (2023). Short and Long-Term Prognostic Significance of Galectin-3 in Patients with ST-Elevation Myocardial Infarction Undergoing Primary Percutaneous Coronary Intervention. Angiology.

[74] Chen H., Chen C., Fang J., Wang R., Nie W. (2020). Circulating galectin-3 on admission and prognosis in acute heart failure patients: A meta-analysis. Heart Fail. Rev..

[75] Cheng W., Fuernau G., Desch S., Freund A., Feistritzer H.J., Pöss J., Besler C., Lurz P., Büttner P., Thiele H. (2022). Circulating Galectin-3 in Patients with Cardiogenic Shock Complicating Acute Myocardial Infarction Treated with Mild Hypothermia: A Biomarker Sub-Study of the SHOCK-COOL Trial. J. Clin. Med..

[76] Wu C., Lv Z., Li X., Zhou X., Mao W., Zhu M. (2021). Galectin-3 in Predicting Mortality of Heart Failure: A Systematic Review and Meta-Analysis. Heart Surg. Forum.

[77] Yao L., Tan Y., Chen F. (2022). Correlation between galectin-3, RDW, Hepc, HS and ferritin and prognosis of patients with acute onset of chronic heart failure. BMC Cardiovasc. Disord..

[78] Bansal N., Zelnick L.R., Soliman E.Z., Anderson A., Christenson R., DeFilippi C., Deo R., Feldman H.I., He J., Ky B. (2021). Change in Cardiac Biomarkers and Risk of Incident Heart Failure and Atrial Fibrillation in CKD: The Chronic Renal Insufficiency Cohort (CRIC) Study. Am. J. Kidney Dis..

[79] Caravaca Perez P., González-Juanatey J.R., Nuche J., Matute-Blanco L., Serrano I., Martínez Selles M., Vázquez García R., Martínez Dolz L., Gómez-Bueno M., Pascual Figal D. (2022). Renal Function Impact in the Prognostic Value of Galectin-3 in Acute Heart Failure. Front. Cardiovasc. Med..

[80] Horiuchi Y.U., Wettersten N., DJ V.A.N.V., Mueller C., Filippatos G., Nowak R., Hogan C., Kontos M.C., Cannon C.M., Müeller G.A. (2023). Galectin-3, Acute Kidney Injury and Myocardial Damage in Patients with Acute Heart Failure. J. Card. Fail..

[81] Horiuchi Y., Wettersten N., van Veldhuisen D.J., Mueller C., Nowak R., Hogan C., Kontos M.C., Cannon C.M., Birkhahn R., Vilke G.M. (2023). The influence of body mass index on clinical interpretation of established and novel biomarkers in acute heart failure. J. Card. Fail..

[82] Hara A., Niwa M., Kanayama T., Noguchi K., Niwa A., Matsuo M., Kuroda T., Hatano Y., Okada H., Tomita H. (2020). Galectin-3: A Potential Prognostic and Diagnostic Marker for Heart Disease and Detection of Early Stage Pathology. Biomolecules.

[83] Shah N.N., Ayyadurai P., Saad M., Kosmas C.E., Dogar M.U., Patel U., Vittorio T.J. (2020). Galactin-3 and soluble ST2 as complementary tools to cardiac MRI for sudden cardiac death risk stratification in heart failure: A review. JRSM Cardiovasc. Dis..

[84] Baccouche B.M., Mahmoud M.A., Nief C., Patel K., Natterson-Horowitz B. (2023). Galectin-3 is Associated with Heart Failure Incidence: A Meta-Analysis. Curr. Cardiol. Rev..

[85] Paulus W.J., Tschöpe C. (2013). A novel paradigm for heart failure with preserved ejection fraction: Comorbidities drive myocardial dysfunction and remodeling through coronary microvascular endothelial inflammation. J. Am. Coll. Cardiol..

[86] Kanagala P., Arnold J.R., Singh A., Chan D.C.S., Cheng A.S.H., Khan J.N., Gulsin G.S., Yang J., Zhao L., Gupta P. (2020). Characterizing heart failure with preserved and reduced ejection fraction: An imaging and plasma biomarker approach. PLoS ONE.

[87] He J., Li X., Luo H., Li T., Zhao L., Qi Q., Liu Y., Yu Z. (2017). Galectin-3 mediates the pulmonary arterial hypertension-induced right ventricular remodeling through interacting with NADPH oxidase 4. J. Am. Soc. Hypertens..

[88] Martin-Saldaña S., Chevalier M.T., Pandit A. (2022). Therapeutic potential of targeting galectins—A biomaterials-focused perspective. Biomaterials.

[89] Felker G.M., Fiuzat M., Shaw L.K., Clare R., Whellan D.J., Bettari L., Shirolkar S.C., Donahue M., Kitzman D.W., Zannad F. (2012). Galectin-3 in ambulatory patients with heart failure: Results from the HF-ACTION study. Circ. Heart Fail..

[90] Atabakhshian R., Kazerouni F., Raygan F., Amirrasouli H., Rahimipour A., Shakeri N. (2014). Assessment of the Relationship between Galectin-3 and Ejection Fraction and Functional Capacity in the Patients with Compensated Systolic Heart Failure. Int. Cardiovasc. Res. J..

[91] Fudim M., Kelly J.P., Jones A.D., AbouEzzeddine O.F., Ambrosy A.P., Greene S.J., Reddy Y.N.V., Anstrom K.J., Alhanti B., Lewis G.D. (2020). Are existing and emerging biomarkers associated with cardiorespiratory fitness in patients with chronic heart failure?. Am. Heart J..

[92] Karolko B., Serafin A., Przewłocka-Kosmala M. (2022). Impact of moderately reduced renal function on the diagnostic and prognostic value of galectin-3 in patients with exertional dyspnea. Adv. Clin. Exp. Med..

[93] Haid M.E., Zylla S., Paulista Markus M.R., Friedrich N., Ewert R., Gläser S., Felix S.B., Dörr M., Bahls M. (2022). Sex-specific associations of cardiorespiratory fitness and galectin-3 in the general population. ESC Heart Fail..

[94] Fernandes-Silva M.M., Guimaraes G.V., Rigaud V.O., Lofrano-Alves M.S., Castro R.E., de Barros Cruz L.G., Bocchi E.A., Bacal F. (2017). Inflammatory biomarkers and effect of exercise on functional capacity in patients with heart failure: Insights from a randomized clinical trial. Eur. J. Prev. Cardiol..

[95] Billebeau G., Vodovar N., Sadoune M., Launay J.M., Beauvais F., Cohen-Solal A. (2017). Effects of a cardiac rehabilitation programme on plasma cardiac biomarkers in patients with chronic heart failure. Eur. J. Prev. Cardiol..

[96] Avazpour S., Amini A., Shirvani H., Arabzadeh E. (2022). Exercise modulation in inflammation and metabolic hormonal disorders of COVID-19 to decrease risk factors in coronary heart disease. Horm. Mol. Biol. Clin. Investig..

[97] Wu C.K., Su M.Y., Lee J.K., Chiang F.T., Hwang J.J., Lin J.L., Chen J.J., Liu F.T., Tsai C.T. (2015). Galectin-3 level and the severity of cardiac diastolic dysfunction using cellular and animal models and clinical indices. Sci. Rep..

[98] Elsadek A., Ibrahim M., El Fallah A.A., Elian M., Deraz S.E. (2022). Galectin-3 as an early marker of diastolic dysfunction in children with end-stage renal disease on regular hemodialysis. Ann. Pediatr. Cardiol..

[99] Ansari U., Behnes M., Hoffmann J., Natale M., Fastner C., El-Battrawy I., Rusnak J., Kim S.H., Lang S., Hoffmann U. (2018). Galectin-3 Reflects the Echocardiographic Grades of Left Ventricular Diastolic Dysfunction. Ann. Lab. Med..

[100] Shi Y., Dong G., Liu J., Shuang X., Liu C., Yang C., Qing W., Qiao W. (2022). Clinical Implications of Plasma Galectin-3 in Heart Failure With Preserved Ejection Fraction: A Meta-Analysis. Front. Cardiovasc. Med..

[101] Lisowska A., Knapp M., Tycińska A., Motybel E., Kamiński K., Święcki P., Musiał W.J., Dymicka-Piekarska V. (2016). Predictive value of Galectin-3 for the occurrence of coronary artery disease and prognosis after myocardial infarction and its association with carotid IMT values in these patients: A mid-term prospective cohort study. Atherosclerosis.

[102] Lok D.J., Lok S.I., Bruggink-André de la Porte P.W., Badings E., Lipsic E., van Wijngaarden J., de Boer R.A., van Veldhuisen D.J., van der Meer P. (2013). Galectin-3 is an independent marker for ventricular remodeling and mortality in patients with chronic heart failure. Clin. Res. Cardiol..

[103] Zaborska B., Sygitowicz G., Smarż K., Pilichowska-Paszkiet E., Budaj A. (2020). Galectin-3 is related to right ventricular dysfunction in heart failure patients with reduced ejection fraction and may affect exercise capacity. Sci. Rep..

[104] Shah R.V., Chen-Tournoux A.A., Picard M.H., van Kimmenade R.R., Januzzi J.L. (2010). Galectin-3, cardiac structure and function, and long-term mortality in patients with acutely decompensated heart failure. Eur. J. Heart Fail..

[105] Screever E.M., Gorter T.M., Willems T.P., Aboumsallem J.P., Suthahar N., Mahmoud B., van Veldhuisen D.J., de Boer R.A., Meijers W.C. (2023). Diffuse Myocardial Fibrosis on Cardiac Magnetic Resonance Imaging Is Related to Galectin-3 and Predicts Outcome in Heart Failure. Biomolecules.

[106] Jan A., Dawkins I., Murphy N., Collier P., Baugh J., Ledwidge M., McDonald K., Watson C.J. (2013). Associates of an elevated natriuretic peptide level in stable heart failure patients: Implications for targeted management. Sci. World J..

[107] Watson C.J., Gallagher J., Wilkinson M., Russell-Hallinan A., Tea I., James S., O’Reilly J., O’Connell E., Zhou S., Ledwidge M. (2021). Biomarker profiling for risk of future heart failure (HFpEF) development. J. Transl. Med..

[108] Huttin O., Kobayashi M., Ferreira J.P., Coiro S., Bozec E., Selton-Suty C., Filipetti L., Lamiral Z., Rossignol P., Zannad F. (2021). Circulating multimarker approach to identify patients with preclinical left ventricular remodelling and/or diastolic dysfunction. ESC Heart Fail..

[109] Lorenzo-Almorós A., Pello A., Aceña Á., Martínez-Milla J., González-Lorenzo Ó., Tarín N., Cristóbal C., Blanco-Colio L.M., Martín-Ventura J.L., Huelmos A. (2020). Galectin-3 Is Associated with Cardiovascular Events in Post-Acute Coronary Syndrome Patients with Type-2 Diabetes. J. Clin. Med..

[110] Bozkurt B., Coats A.J., Tsutsui H., Abdelhamid M., Adamopoulos S., Albert N., Anker S.D., Atherton J., Böhm M., Butler J. (2021). Universal Definition and Classification of Heart Failure: A Report of the Heart Failure Society of America, Heart Failure Association of the European Society of Cardiology, Japanese Heart Failure Society and Writing Committee of the Universal Definition of Heart Failure. J. Card. Fail..

[111] Mohebi R., Murphy S., Jackson L., McCarthy C., Abboud A., Murtagh G., Gawel S., Miksenas H., Gaggin H., Januzzi J.L. (2022). Biomarker prognostication across Universal Definition of Heart Failure stages. ESC Heart Fail..

[112] Trippel T.D., Mende M., Düngen H.D., Hashemi D., Petutschnigg J., Nolte K., Herrmann-Lingen C., Binder L., Hasenfuss G., Pieske B. (2021). The diagnostic and prognostic value of galectin-3 in patients at risk for heart failure with preserved ejection fraction: Results from the DIAST-CHF study. ESC Heart Fail..

[113] Srivatsan V., George M., Shanmugam E. (2015). Utility of galectin-3 as a prognostic biomarker in heart failure: Where do we stand?. Eur. J. Prev. Cardiol..

[114] McDowell K., Campbell R., Simpson J., Cunningham J.W., Desai A.S., Jhund P.S., Lefkowitz M.P., Rouleau J.L., Swedberg K., Zile M.R. (2023). Incremental prognostic value of biomarkers in PARADIGM-HF. Eur. J. Heart Fail..

[115] Lopez-Andrès N., Rossignol P., Iraqi W., Fay R., Nuée J., Ghio S., Cleland J.G., Zannad F., Lacolley P. (2012). Association of galectin-3 and fibrosis markers with long-term cardiovascular outcomes in patients with heart failure, left ventricular dysfunction, and dyssynchrony: Insights from the CARE-HF (Cardiac Resynchronization in Heart Failure) trial. Eur. J. Heart Fail..

[116] Stolen C.M., Adourian A., Meyer T.E., Stein K.M., Solomon S.D. (2014). Plasma galectin-3 and heart failure outcomes in MADIT-CRT (multicenter automatic defibrillator implantation trial with cardiac resynchronization therapy). J. Card. Fail..

[117] Zaborska B., Pilichowska-Paszkiet E., Makowska E., Sygitowicz G., Słomski T., Zaborski M., Budaj A. (2021). Prognostic value of galectin-3 and right ventricular function for long-term mortality in heart failure patients treated with cardiac resynchronization therapy. Sci. Rep..

[118] Crnkovic S., Egemnazarov B., Damico R., Marsh L.M., Nagy B.M., Douschan P., Atsina K., Kolb T.M., Mathai S.C., Hooper J.E. (2019). Disconnect between Fibrotic Response and Right Ventricular Dysfunction. Am. J. Respir. Crit. Care Med..

[119] Shen Q., Chen W., Liu J., Liang Q. (2019). Galectin-3 aggravates pulmonary arterial hypertension via immunomodulation in congenital heart disease. Life Sci..

[120] Hao M., Li M., Li W. (2017). Galectin-3 inhibition ameliorates hypoxia-induced pulmonary artery hypertension. Mol. Med. Rep..

[121] Sohns C., Marrouche N.F. (2020). Atrial fibrillation and cardiac fibrosis. Eur. Heart J..

[122] Webber M., Jackson S.P., Moon J.C., Captur G. (2020). Myocardial Fibrosis in Heart Failure: Anti-Fibrotic Therapies and the Role of Cardiovascular Magnetic Resonance in Drug Trials. Cardiol. Ther..

[123] Hindricks G., Potpara T., Dagres N., Arbelo E., Bax J.J., Blomström-Lundqvist C., Boriani G., Castella M., Dan G.A., Dilaveris P.E. (2021). 2020 ESC Guidelines for the diagnosis and management of atrial fibrillation developed in collaboration with the European Association for Cardio-Thoracic Surgery (EACTS): The Task Force for the diagnosis and management of atrial fibrillation of the European Society of Cardiology (ESC) Developed with the special contribution of the European Heart Rhythm Association (EHRA) of the ESC. Eur. Heart J..

[124] Sartipy U., Dahlström U., Fu M., Lund L.H. (2017). Atrial Fibrillation in Heart Failure With Preserved, Mid-Range, and Reduced Ejection Fraction. JACC Heart Fail..

[125] Goldberger J.J., Arora R., Green D., Greenland P., Lee D.C., Lloyd-Jones D.M., Markl M., Ng J., Shah S.J. (2015). Evaluating the Atrial Myopathy Underlying Atrial Fibrillation: Identifying the Arrhythmogenic and Thrombogenic Substrate. Circulation.

[126] Goette A., Kalman J.M., Aguinaga L., Akar J., Cabrera J.A., Chen S.A., Chugh S.S., Corradi D., D’Avila A., Dobrev D. (2017). EHRA/HRS/APHRS/SOLAECE expert consensus on atrial cardiomyopathies: Definition, characterization, and clinical implication. Heart Rhythm.

[127] Sygitowicz G., Maciejak-Jastrzębska A., Sitkiewicz D. (2021). A Review of the Molecular Mechanisms Underlying Cardiac Fibrosis and Atrial Fibrillation. J. Clin. Med..

[128] Sonmez O., Ertem F.U., Vatankulu M.A., Erdogan E., Tasal A., Kucukbuzcu S., Goktekin O. (2014). Novel fibro-inflammation markers in assessing left atrial remodeling in non-valvular atrial fibrillation. Med. Sci. Monit..

[129] Gurses K.M., Yalcin M.U., Kocyigit D., Canpinar H., Evranos B., Yorgun H., Sahiner M.L., Kaya E.B., Ozer N., Tokgozoglu L. (2015). Effects of persistent atrial fibrillation on serum galectin-3 levels. Am. J. Cardiol..

[130] Yalcin M.U., Gurses K.M., Kocyigit D., Canpinar H., Canpolat U., Evranos B., Yorgun H., Sahiner M.L., Kaya E.B., Hazirolan T. (2015). The Association of Serum Galectin-3 Levels with Atrial Electrical and Structural Remodeling. J. Cardiovasc. Electrophysiol..

[131] Hernández-Romero D., Vílchez J.A., Lahoz Á., Romero-Aniorte A.I., Jover E., García-Alberola A., Jara-Rubio R., Martínez C.M., Valdés M., Marín F. (2017). Galectin-3 as a marker of interstitial atrial remodelling involved in atrial fibrillation. Sci. Rep..

[132] Nezami Z., Holm H., Ohlsson M., Molvin J., Korduner J., Bachus E., Zaghi A., Dieden A., Platonov P.G., Jujic A. (2022). The impact of myocardial fibrosis biomarkers in a heart failure population with atrial fibrillation-The HARVEST-Malmö study. Front. Cardiovasc. Med..

[133] Aksan G., Yanık A., Yontar O.C., Gedikli Ö., Arslan U., Soylu K. (2021). Galectin-3 levels and the prediction of atrial high-rate episodes in patients with cardiac resynchronization therapy. J. Investig. Med..

[134] Mosleh W., Chaudhari M.R., Sonkawade S., Mahajan S., Khalil C., Frodey K., Shah T., Dahal S., Karki R., Katkar R. (2018). The Therapeutic Potential of Blocking Galectin-3 Expression in Acute Myocardial Infarction and Mitigating Inflammation of Infarct Region: A Clinical Outcome-Based Translational Study. Biomark. Insights.

[135] Al-Salam S., Hashmi S. (2018). Myocardial Ischemia Reperfusion Injury: Apoptotic, Inflammatory and Oxidative Stress Role of Galectin-3. Cell. Physiol. Biochem..

[136] Nguyen M.N., Ziemann M., Kiriazis H., Su Y., Thomas Z., Lu Q., Donner D.G., Zhao W.B., Rafehi H., Sadoshima J. (2019). Galectin-3 deficiency ameliorates fibrosis and remodeling in dilated cardiomyopathy mice with enhanced Mst1 signaling. Am. J. Physiol. Heart Circ. Physiol..

[137] Yu L., Ruifrok W.P., Meissner M., Bos E.M., van Goor H., Sanjabi B., van der Harst P., Pitt B., Goldstein I.J., Koerts J.A. (2013). Genetic and pharmacological inhibition of galectin-3 prevents cardiac remodeling by interfering with myocardial fibrogenesis. Circ. Heart Fail..

[138] Böcker S., Laaf D., Elling L. (2015). Galectin Binding to Neo-Glycoproteins: LacDiNAc Conjugated BSA as Ligand for Human Galectin-3. Biomolecules.

[139] Xu G.R., Zhang C., Yang H.X., Sun J.H., Zhang Y., Yao T.T., Li Y., Ruan L., An R., Li A.Y. (2020). Modified citrus pectin ameliorates myocardial fibrosis and inflammation via suppressing galectin-3 and TLR4/MyD88/NF-κB signaling pathway. Biomed. Pharmacother..

[140] Gao X., Zhi Y., Zhang T., Xue H., Wang X., Foday A.D., Tai G., Zhou Y. (2012). Analysis of the neutral polysaccharide fraction of MCP and its inhibitory activity on galectin-3. Glycoconj. J..

[141] Calvier L., Martinez-Martinez E., Miana M., Cachofeiro V., Rousseau E., Sádaba J.R., Zannad F., Rossignol P., López-Andrés N. (2015). The impact of galectin-3 inhibition on aldosterone-induced cardiac and renal injuries. JACC Heart Fail..

[142] Gao X., Zhi Y., Sun L., Peng X., Zhang T., Xue H., Tai G., Zhou Y. (2013). The inhibitory effects of a rhamnogalacturonan I (RG-I) domain from ginseng pectin on galectin-3 and its structure-activity relationship. J. Biol. Chem..

[143] Pozder Geb Gehlken C., Rogier van der Velde A., Meijers W.C., Sillje H.H.W., Muntendam P., Dokter M.M., van Gilst W.H., Schols H.A., de Boer R.A. (2022). Pectins from various sources inhibit galectin-3-related cardiac fibrosis. Curr. Res. Transl. Med..

[144] Hirani N., MacKinnon A.C., Nicol L., Ford P., Schambye H., Pedersen A., Nilsson U.J., Leffler H., Sethi T., Tantawi S. (2021). Target inhibition of galectin-3 by inhaled TD139 in patients with idiopathic pulmonary fibrosis. Eur. Respir. J..

[145] Slack R.J., Mills R., Mackinnon A.C. (2021). The therapeutic potential of galectin-3 inhibition in fibrotic disease. Int. J. Biochem. Cell Biol..

[146] Kirk J.A., Frangogiannis N.G. (2018). Galectin-3 in the pathogenesis of heart failure: A causative mediator or simply a biomarker?. Am. J. Physiol. Heart Circ. Physiol..

[147] Edelmann F., Holzendorf V., Wachter R., Nolte K., Schmidt A.G., Kraigher-Krainer E., Duvinage A., Unkelbach I., Düngen H.D., Tschöpe C. (2015). Galectin-3 in patients with heart failure with preserved ejection fraction: Results from the Aldo-DHF trial. Eur. J. Heart Fail..

[148] Cleland J.G.F., Ferreira J.P., Mariottoni B., Pellicori P., Cuthbert J., Verdonschot J.A.J., Petutschnigg J., Ahmed F.Z., Cosmi F., Brunner La Rocca H.P. (2021). The effect of spironolactone on cardiovascular function and markers of fibrosis in people at increased risk of developing heart failure: The heart ‘Omics’ in AGEing (HOMAGE) randomized clinical trial. Eur. Heart J..

[149] De Marco C., Claggett B.L., de Denus S., Zile M.R., Huynh T., Desai A.S., Sirois M.G., Solomon S.D., Pitt B., Rouleau J.L. (2021). Impact of diabetes on serum biomarkers in heart failure with preserved ejection fraction: Insights from the TOPCAT trial. ESC Heart Fail..

[150] Tran S.K., Ngo T.H., Lai T.T., Truong G.K., Tran K.D.D., Vo P.M., Nguyen P.T., Nguyen P.H., Nguyen T.T., Nguyen O.T.K. (2023). Effectiveness of Spironolactone in Terms of Galectin-3 Levels in Patients with Heart Failure with a Reduced Ejection Fraction in the Vietnamese Population. Healthcare.

[151] Du X.J., Zhao W.B., Nguyen M.N., Lu Q., Kiriazis H. (2019). β-Adrenoceptor activation affects galectin-3 as a biomarker and therapeutic target in heart disease. Br. J. Pharmacol..

[152] Zhao W.B., Lu Q., Nguyen M.N., Su Y., Ziemann M., Wang L.N., Kiriazis H., Puthalakath H., Sadoshima J., Hu H.Y. (2019). Stimulation of β-adrenoceptors up-regulates cardiac expression of galectin-3 and BIM through the Hippo signalling pathway. Br. J. Pharmacol..

[153] Agnello L., Bellia C., Lo Sasso B., Pivetti A., Muratore M., Scazzone C., Bivona G., Lippi G., Ciaccio M. (2017). Establishing the upper reference limit of Galectin-3 in healthy blood donors. Biochem. Med..

[154] Mueller T., Egger M., Leitner I., Gabriel C., Haltmayer M., Dieplinger B. (2016). Reference values of galectin-3 and cardiac troponins derived from a single cohort of healthy blood donors. Clin. Chim. Acta.

[155] Lima T., Perpétuo L., Henrique R., Fardilha M., Leite-Moreira A., Bastos J., Vitorino R. (2023). Galectin-3 in prostate cancer and heart diseases: A biomarker for these two frightening pathologies?. Mol. Biol. Rep..

[156] Sciacchitano S., Lavra L., Morgante A., Ulivieri A., Magi F., De Francesco G.P., Bellotti C., Salehi L.B., Ricci A. (2018). Galectin-3: One Molecule for an Alphabet of Diseases, from A to Z. Int. J. Mol. Sci..

[157] Zhu L., Li N., Sun L., Zheng D., Shao G. (2021). Non-coding RNAs: The key detectors and regulators in cardiovascular disease. Genomics.

[158] Wang Y., Sun X. (2020). The functions of LncRNA in the heart. Diabetes Res. Clin. Pract..

[159] Sygitowicz G., Tomaniak M., Błaszczyk O., Kołtowski Ł., Filipiak K.J., Sitkiewicz D. (2015). Circulating microribonucleic acids miR-1, miR-21 and miR-208a in patients with symptomatic heart failure: Preliminary results. Arch. Cardiovasc. Dis..

[160] Sygitowicz G., Sitkiewicz D. (2022). Involvement of circRNAs in the Development of Heart Failure. Int. J. Mol. Sci..

